# Case report: Success of tepotinib therapy in overcoming resistance to osimertinib in a patient with EGFR-mutant lung adenocarcinoma with a potential acquired MET exon 14 skipping mutation

**DOI:** 10.3389/fonc.2022.965741

**Published:** 2022-10-13

**Authors:** Shinkichi Takamori, Takashi Seto, Masafumi Yamaguchi, Fumihiko Kinoshita, Takatoshi Fujishita, Kensaku Ito, Ryo Toyozawa, Fumihiro Shoji, Tatsuro Okamoto

**Affiliations:** ^1^ Department of Thoracic Oncology, National Hospital Organization Kyushu Cancer Center, Fukuoka, Japan; ^2^ Department of Surgery and Science, Graduate School of Medical Sciences, Kyushu University, Fukuoka, Japan; ^3^ Department of Thoracic Surgery, Kitakyushu Municipal Medical Center, Fukuoka, Japan

**Keywords:** lung adenocarcinoma, *MET*ex14del, tepotinib, osimertinib, resistance

## Abstract

Osimertinib is a standard therapy for the treatment of advanced non-small cell lung cancer (NSCLC) harboring epidermal growth factor receptor gene (*EGFR*) mutations, but most patients with *EGFR*-mutant NSCLC develop secondary resistance to osimertinib. Mesenchymal-epithelial transition gene (*MET*) alterations and oncogene fusions have been identified as the most common mechanisms of resistance to osimertinib. However, *MET* exon 14 skipping mutation (*MET*ex14del) as an acquired resistance to osimertinib has rarely been reported. A non-smoking 76-year-old woman was diagnosed with lung adenocarcinoma in the right lower lobe (cT2bN2M1c [pulmonary and bone metastases], cStage IVB). The primary tumor was submitted to cobas^®^ EGFR Mutation Test v2 (Roche Diagnostics Ltd.), next generation sequencing (Oncomine Comprehensive Assay v3; Thermo Fisher Scientific), the AmoyDx^®^ Essential NGS panel (Amoy Diagnostics, Xiamen, China), all of which were positive for *EGFR L858R* and *de novo T790M*. We administered daily osimertinib (80 mg/day), and achieved a partial response. However, after 14.0 months, computed tomography showed progression of the primary tumor and lung metastases. Re-biopsy of the primary tumor was conducted, and the specimen was submitted to Archer^®^MET companion diagnostic for detection of *MET*ex14del. Although the primary tumor was negative for *MET*ex14del, the re-biopsy specimen was positive for *MET*ex14del. We validated that the biopsy specimen of the primary tumor at diagnosis before osimertinib administration was negative for *MET*ex14del using local reverse transcription PCR. We administered daily tepotinib (500 mg/day) to the patient as a further-line treatment, and achieved a partial response (tumor shrinkage rate: 34.5%) after 2.0 months, who responded to tepotinib therapy for 8.0 months. We described a patient with lung adenocarcinoma harboring *MET*ex14del as a potential acquired resistance to osimertinib, who responded to subsequent tepotinib therapy. Re-biopsy and re-analysis of genetic profiles should be considered in NSCLC patients who develop osimertinib resistance.

## Introduction

Epidermal growth factor receptor gene (EGFR)-tyrosine kinase inhibitors (TKIs) are approved by the United States Food and Drug Administration for the treatment of advanced non-small cell lung cancer (NSCLC) harboring *EGFR* mutations (1). Osimertinib (Tagrisso™, [AZD9291] AstraZeneca, Cambridge, UK), a third-generation, irreversible, EGFR-TKI, is an approved therapy (80 mg, once-daily) for treatment of patients with metastatic *EGFR*-mutated NSCLC and those with T790M-positive NSCLC after disease progression on EGFR-TKIs (1, 2). However, despite very high objective response rates, most patients with *EGFR*-mutant NSCLC develop secondary resistance to EGFR-TKIs. Secondary resistance can be broadly classified into *EGFR*-dependent and independent mechanisms (3). Mesenchymal-epithelial transition gene (*MET*) alterations, *EGFR* C797X, small cell lung cancer transformation, and oncogene fusions have previously been identified as the most common mechanisms of resistance to EGFR-TKIs (3).

*MET* exon 14 skipping mutation (*MET*ex14del), a splice-site oncogenic mutation, is found in 2%–3% of NSCLC patients (4). Such patients respond well to MET-tyrosine kinase inhibitors, including tepotinib (5). Tepotinib is a once-daily oral type Ib (highly selective) MET inhibitor, blocking ATP binding to prevent phosphorylation and activation of the MET receptor. Tepotinib has shown promising clinical activity in patients with NSCLC harboring METex14del, with the response rate of 46% (95% confidence interval, 36 to 57) and a median duration of response of 11.1 months (5). Although *MET* amplification has already been reported from the viewpoint of secondary resistance to EGFR-TKIs, *MET*ex14del has rarely been documented (3, 6). We herein report a rare case of a patient with lung adenocarcinoma harboring the *MET*ex14del mutation as a potential acquired resistance to osimertinib, who responded to subsequent tepotinib therapy.

## Case report

A non-smoking 76-year-old woman was diagnosed with pulmonary nodules following a cancer screening. Several radiological examinations and bronchial biopsy led to a diagnosis of primary lung adenocarcinoma in the right lower lobe (cT2bN2M1c [pulmonary and bone metastases], cStage IVB according to the 8th Edition of TNM in Lung Cancer). The primary tumor was submitted to cobas^®^ EGFR Mutation Test v2 (Roche Diagnostics Ltd.), next generation sequencing (Oncomine Comprehensive Assay v3; Thermo Fisher Scientific), and the AmoyDx^®^ Essential NGS panel (Amoy Diagnostics, Xiamen, China), all of which were positive for *EGFR L858R* and *de novo T790M*. We administered daily osimertinib (80 mg/day), and achieved a partial response. However, after 14.0 months, chest computed tomography (CT) showed progression of the primary tumor and lung metastases.

**Figure 1:**
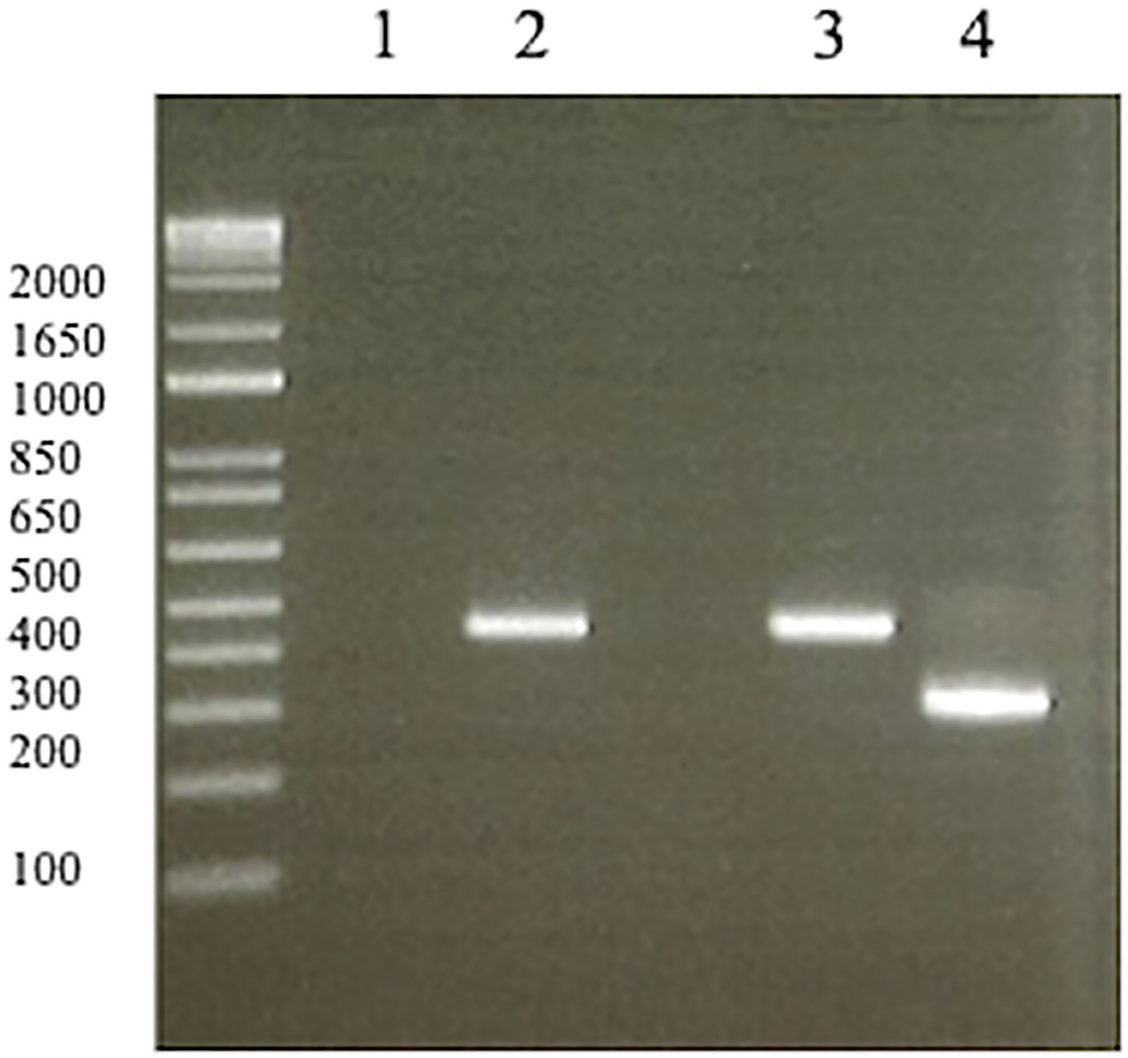
Reverse transcription PCR of negative/positive controls (1, 3, 4) and biopsy specimen before osimertinib administration from the patient (2). (1) No template (negative control). (2) The biopsy specimen before osimertinib administration was negative for *MET*ex14del. (3) *MET*ex14del wild type sample (negative control). (4) *MET*ex14del positive sample (positive control). *MET*, mesenchymal-epithelial transition gene.

As a second-line therapy, pemetrexed (500 mg/m^2^) had been administered for 6.7 months. The CT scan subsequently showed right femoral bone metastasis, and palliative radiation therapy (30 Gy/10 Fr) was administered because of the pain. Re-biopsy of the primary tumor was conducted, and the specimen was submitted to Archer^®^MET companion diagnostic for detection of *MET*ex14del, which is a companion diagnostic for tepotinib. This was positive for *MET*ex14del. The re-biopsy specimen was positive for *EGFR L858R* and *de novo T790M*, although the biopsy specimen of the primary tumor before osimertinib administration was negative for *MET*ex14del using local reverse transcription PCR (**Figure 1**).

**Figure 2:**
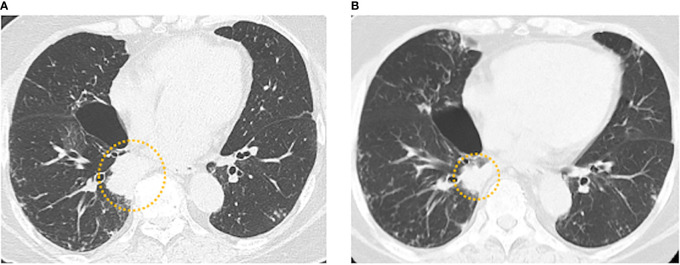
**(A)** Baseline chest computed tomography (CT) before tepotinib administration showing the tumor in the right lower lobe. **(B)** Chest CT 2.0 months after tepotinib administration showing a partial response (tumor shrinkage rate: 34.5% in diameter).

We administered daily tepotinib (500 mg/day) to the patient as a further-line therapy, and achieved a partial response (tumor shrinkage rate: 34.5% in diameter) after 2.0 months (**Figures 2A, B**). Although the tumor had been responded to tepotinib therapy for 8.0 months, CT and positron emission tomography revealed left femoral bone metastasis as a new lesion. After subsequent therapies (nab-paclitaxel [100 mg/m^2^ on days 1, 8, 15] for 1.3 months and S-1 [100 mg/body on days 1-14] for 1.0 month), the patient died because of disease progression 32.3 months after administration of first-line osimertinib therapy.

## Discussion

We herein report a rare case of a patient with lung adenocarcinoma harboring *MET*ex14del following resistance to osimertinib, who responded to subsequent tepotinib therapy. We have identified two possible hypotheses that could explain this phenomenon. First, *MET*ex14del was not present before osimertinib therapy, but was induced by osimertinib as an acquired resistance. Second, *MET*ex14del was present at very low copy numbers in the lung adenocarcinoma, and was not detected in the original biopsy because of tumor heterogeneity, suggesting the coexistence of *EGFR* and *MET*ex14del mutations.

With regard to *MET*ex14del as an acquired resistance, a few studies have reported similar findings (6, 7). Pinquie F et al. reported a case of treatment with crizotinib to overcome resistance to osimertinib in an *EGFR*-mutant NSCLC patient harboring an acquired *MET*ex14del. In that report, the CT-scan after 4 months crizotinib of monotherapy showed a complete response of lung metastases, progression of liver metastases, and reappearance of pleural nodules. Thus, the clinical activity of crizotinib was heterogeneous, and crizotinib monotherapy could not confirm a partial response. In our report, a partial response was achieved with tepotinib therapy, and the duration of response was 8.0 months. The discrepancy in efficacy may be due to the difference in types of MET-TKIs. Crizotinib is a type Ia inhibitor, blocking ATP binding to prevent phosphorylation/activation of the receptor, whereas tepotinib is a type Ib inhibitor, blocking MET more specifically than type Ia inhibitors (8). According to preclinical work by Suzuwa et al. (7), cell viability assays using an EGFR-mutant NSCLC cell model transfected with a lentiviral vector expressing *MET*ex14del revealed that *MET*ex14del reduced sensitivity to osimertinib, and *MET*ex14del expression correlated with upregulation of phosphorylated *EGFR*. These results indicated that *MET*ex14del induces resistance to osimertinib in *EGFR*-mutant NSCLC cells. Suzuwa et al. also reported that crizotinib monotherapy only inhibited *MET*ex14del phosphorylation in EGFR-mutant and *MET*ex14del-transfected NSCLC cell, and did not block phosphorylation of EGFR, AKT, and ERK, which suggests that EGFR is still signaling (7). Accordingly, a combination therapy of osimertinib and MET inhibitors was effective against *MET*ex14del-induced drug resistance in *EGFR*-mutant NSCLC cells. The treatment of patients with two driver oncogene mutations is thought to require the inhibition of both regulatory pathways, which is being investigated in ongoing clinical trials (9, 10). Regarding the coexistence of *EGFR* and *MET*ex14del mutations, a previous study reported concomitant *EGFR* and *MET*ex14del frequencies of 0.2% (three out of 1,590 cases in a Chinese cohort) (11). There is a possibility that *MET*ex14del was present at very low copy numbers in the original biopsy specimen, but we confirmed that the biopsy specimen of the primary tumor before osimertinib administration was negative for *MET*ex14del using local reverse transcription PCR. In any case, re-biopsy and re-analysis of genetic profiles in NSCLC should be considered after osimertinib resistance.

In our patient, the duration of the response to tepotinib (8.0 months) was slightly shorter than the median duration of response reported in a previous clinical trial (11.1 months) (5). This may be because *de novo* and acquired *MET*ex14del differ in their response to tepotinib, and/or because *EGFR* and *MET*ex14del co-mutations require both EGFR- and MET-TKI therapies (12). Nevertheless, the duration of response to tepotinib is expected to be longer than that of chemotherapy in the treatment of lung adenocarcinoma with *EGFR* and *MET*ex14del mutations. Therefore, we recommend that re-biopsy and re-analysis of genetic profiles should be considered in NSCLC patients who have developed osimertinib resistance.

## Conclusion

We report a rare case of a patient with lung adenocarcinoma harboring *MET*ex14del as a potential acquired resistance to osimertinib, who responded to tepotinib therapy. Re-biopsy and re-analysis of genetic profiles in NSCLC should be considered in cases of osimertinib resistance.

## Data availability statement

The datasets for this article are not publicly available due to concerns regarding participant/patient anonymity. Requests to access the datasets should be directed to the corresponding author.

## Ethics statement

The studies involving human participants were reviewed and approved by the institutional review boards of National Hospital Organization Kyushu Cancer Center (IRB No. 2019-45). Written informed consent for publication was obtained from the legally authorized representative of the patient.

## Author contributions

ST treated the patient and wrote the manuscript. TS significantly contributed to all of the ideas and methods. MY and TO assisted and supervised all of the analyses conducted in this study. FK assisted in drafting the manuscript. TF and FS treated the patient. KI and RT supervised the writing of the manuscript. All authors contributed to the article and approved the submitted version.

## Acknowledgment

We thank Sarah Williams, PhD, from Edanz (https://jp.edanz.com/ac) for editing a draft of this manuscript.

## Conflict of interest

The authors declare that the research was conducted in the absence of any commercial or financial relationships that could be construed as a potential conflict of interest.

## Publisher’s note

All claims expressed in this article are solely those of the authors and do not necessarily represent those of their affiliated organizations, or those of the publisher, the editors and the reviewers. Any product that may be evaluated in this article, or claim that may be made by its manufacturer, is not guaranteed or endorsed by the publisher.
